# Metagenome-Assembled Genome Sequences of Raphidiopsis raciborskii and Planktothrix agardhii from a Cyanobacterial Bloom in Kissena Lake, New York, USA

**DOI:** 10.1128/MRA.01380-20

**Published:** 2021-01-14

**Authors:** Robbie M. Martin, Michael Kausch, Kimarie Yap, John D. Wehr, Gregory L. Boyer, Steven W. Wilhelm

**Affiliations:** a Department of Microbiology, University of Tennessee, Knoxville, Tennessee, USA; b Department of Biological Sciences, Fordham University, Bronx, New York City, New York, USA; c Department of Chemistry, State University of New York College of Environmental Science and Forestry, Syracuse, New York, USA; Georgia Institute of Technology

## Abstract

Raphidiopsis raciborskii and Planktothrix agardhii are filamentous, potentially toxin-producing cyanobacteria that form nuisance blooms in fresh waters. Here, we report high-quality metagenome-assembled genome sequences of *R. raciborskii* and *P. agardhii* collected from a bloom in Kissena Lake, New York.

## ANNOUNCEMENT

Raphidiopsis raciborskii (formerly *Cylindrospermopsis raciborskii*) (1) is a filamentous, nitrogen-fixing cyanobacterium that forms nuisance blooms in fresh waters and is considered an invasive species in temperate ecosystems (2). Its geographic range has expanded in recent decades due to rising lake temperatures and its tolerance of diverse light and nutrient conditions (2). Planktothrix agardhii is a filamentous cyanobacterium found in eutrophic, polymictic lakes of temperate zones. Some strains produce microcystin (3). Both are species of concern in North American lakes (4, 5), yet genome sequences from North American strains are underrepresented in publicly available nucleotide archives. Here, we present metagenome-assembled genome sequences of *R. raciborskii* and *P. agardhii* from Kissena Lake, NY.

Kissena Lake is a 3.4-ha natural lake located in Queens, NY (40.748°N, 73.806°W). Kissena Lake is subject to dense cyanobacterial populations through most of the summer months. Samples were collected on 26 September 2019 during a cyanobacterial bloom microscopically determined as dominated by *R. raciborskii*. From a shoreline position, multiple casts of a phytoplankton net were used to concentrate 1.5 liters of surface seston. The seston was transported to the lab on ice. Two samples (KL1 and KL2) were collected by filtering 300 ml of seston each onto 0.4-μm-pore-size polycarbonate filters. The filters were immediately stored at −20°C.

Total DNA was extracted using a ZymoBIOMICS DNA/RNA miniprep kit according to the manufacturer’s instructions. DNA libraries were prepared and sequenced by the Microbial Genome Sequencing Center, LLC (Pittsburgh, PA). Libraries were prepared using the Illumina Nextera kit and sequenced on the Illumina NextSeq 550 genome sequencer, producing 150-bp paired-end reads. Sequencing produced a total of 14,107,668 (KL1) and 13,236,752 (KL2) reads.

The reads were trimmed with CLC Genomics Workbench v10.1.1 using default settings. metaSPAdes, MaxBin2, CheckM, and FastANI were implemented in KBase using default parameters unless noted (6–10). *De novo* assembly was conducted with metaSPAdes v3.13.0 using a minimum contig length of 2,000 bp. MaxBin2 v2.2.4 was used to recover metagenome-assembled genomes (MAGs). MAG quality was evaluated with CheckM v1.0.18. The sequencing depth of each MAG was estimated by mapping source reads to their respective MAGs. The genomes were annotated using the NCBI Prokaryotic Genome Annotation Pipeline (PGAP) v4.13 (11). Taxonomy was assigned by average nucleotide identity (ANI) comparison calculated by FastANI v0.1.2.

From KL1, we recovered a MAG of *Raphidiopsis raciborskii* comprised of 100 contigs with a combined length of 3,657,391 bp and 40.1% GC content. The sequencing depth was ∼260-fold. CheckM indicated that the genome was 99.7% complete with 0.3% contamination. The ANI was 99% with *R. raciborskii* UTEX LB2897 (USA), 96% with strains from Brazil (ITEP-A1, CYLP, CYRF), and 94% with CS-505 (Australia). PGAP predicted 3,208 coding DNA sequences (CDS), 3,115 proteins, 38 tRNAs, and 4 noncoding RNAs (ncRNAs). From KL2, we recovered a MAG of Planktothrix agardhii comprised of 334 contigs with a combined length of 4,765,279 bp and 39.6% GC content. The sequencing depth was ∼17-fold. CheckM indicated the genome to be 99.6% complete with 0.65% contamination. The ANI was 97% with *P. agardhii* NIVA-CYA 15 (Norway). PGAP predicted 4,402 CDS, 4,285 proteins, 37 tRNAs, and 4 rRNAs.

### Data availability.

The sequences are deposited at DDBJ/ENA/GenBank under the accession no. JADQCS000000000 and JADQCT000000000. The reads are deposited at the NCBI Sequence Read Archive under the accession no. PRJNA675899.
